# Potential nephroprotective effects of angiotensin II type 2 receptor agonist Compound 21 in renal ischemia-reperfusion injury

**DOI:** 10.25122/jml-2023-0120

**Published:** 2023-09

**Authors:** Lafta Fayez Kadhim, Sarmad Nory Gany, Heider Qassam, Najah Rayish Hadi, Salim Kadhim

**Affiliations:** 1 Ministry of Health, Al-Najaf Health Directorate, Al-Najaf, Iraq; 2 Faculty of Medicine, University of Kufa, Al-Najaf, Iraq; 3 Department of Pharmacology & Therapeutics, Faculty of Medicine, University of Kufa, Kufa, Iraq; 4 College of Pharmacy, Al-Kafeel University, Al-Najaf, Iraq

**Keywords:** Compound 21, acute kidney injury, renal ischemia-reperfusion injury, mice, PI3K, AKI: Acute Kidney Injury, ELISA: Enzyme-Linked Immunosorbent Assay, I/R: Ischemia-Reperfusion Injury, P-AKT: Phosphorylated Protein Kinase B, PI3K: Phosphatidylinositol-3-Kinase, IHC: Immunohistochemistry, PCR: Polymerase Chain Reaction

## Abstract

This study examined the reno-protective potential of Compound 21 during renal ischemia-reperfusion injury by regulating the PI3K expression. 20 adult male Swiss-albino mice, aged 8-12 weeks and weighing 20-30g, were randomly assigned to four equal groups: sham, control, vehicle, and Compound 21. Serum urea, creatinine, inflammatory mediators, tissue 8-isoprostane, and myeloperoxidase were quantified using ELISA. Compared to the sham group, blood levels of urea, creatinine, TNF-α, IL-6, and IL-10 were significantly higher in the ischemia-reperfusion group than in the sham group (p<0.05). However, these indicators were significantly lower in the Compound 21 group (p<0.05). Histological analysis revealed significant renal tissue damage in the ischemia-reperfusion group (p<0.05), which was significantly reduced in the Compound 21 group (p<0.05). PCR results showed that PI3K expression was significantly lower (p<0.05) in the control group compared to the sham group but significantly higher in the Compound 21 group (p<0.05). Furthermore, P-AKT expression levels in the control group were considerably lower than in the sham group (p<0.05). On the other hand, the level of P-AKT expression in the Compound 21 group was significantly upregulated compared to the control group (p<0.05). The findings revealed that Compound 21 could mitigate renal dysfunction induced by ischemia-reperfusion injury in male mice through modulation of the PI3K/AKT signaling pathway, resulting in decreased levels of pro-inflammatory cytokines and renal oxidative stress markers.

## INTRODUCTION

Clinical conditions such as localized necrosis, septic shock, and solid organ transplantation frequently involve ischemia and subsequent reperfusion (I/R), which is an inevitable and harmful event. Ischemia occurs when an insufficient supply of oxygen-rich blood leads to tissue hypoxia, accumulation of cellular waste, nutrient deficiency, and carbon dioxide buildup (hypercapnia), ultimately causing cellular death [1]. This lack of blood supply depletes adenosine triphosphate (ATP), leading to cell injury. Ischemic tissue reperfusion, such as after transplantation, provides oxygen and substrates for tissue regeneration, restoring vitality, and removing harmful metabolites. Blood flow to ischemic tissue accelerates tissue damage by triggering a series of molecular events such as elevated levels of reactive oxygen species, cytokines, and chemokines release and leukocyte infiltration [2].

Renal I/R injury is a clinical condition and one of the causes of acute kidney injury (AKI). AKI is characterized by a sudden decrease in renal function as indicated by markers of renal injury, urea and creatinine, reduced urine output, electrolyte disturbances, and metabolic acid accumulation [3]. While AKI is often associated with renal transplantation, a variety of other clinical situations, including myocardial infarction, surgery, and sepsis, can also lead to this condition [4]. The PI3K-AKT pathway, also known as the phosphatidylinositol 3-kinase (PI3K) - protein kinase B (AKT) pathway, is crucial for cellular viability, playing fundamental roles in regulating cell survival, apoptosis, and proliferation [5]. Numerous studies suggest that pharmaceutical drugs with significant antioxidant and anti-inflammatory action might favorably regulate PI3K/AKT signaling pathways, promoting renoprotective effects [6]. The angiotensin II type 2 receptor (AT2R) agonist, Compound 21 (C21), is a non-peptide molecule with an oral bioavailability of 20-30% and a plasma half-life of 0.5-2.5 hours post intravenous administration [7]. Since its synthesis in 2004, C21 has emerged as a highly selective orally active AT2R agonist, providing a novel tool for investigating the therapeutic potential of AT2R [8].

## MATERIAL AND METHODS

### Animals

Male Swiss albino mice (n=20), aged 8 to 12 weeks and weighing 20 to 30 grams, were obtained from the animal laboratory at the Faculty of Science, University of Kufa, Iraq. Mice were kept in separate cages under standard conditions (25°C, 60-65% humidity, and 12 hours of darkness followed by 12 hours of light) with free access to food and water. Euthanasia for tissue collection was performed under deep anesthesia using ketamine (100mg/kg) and xylazine (10mg/kg) to minimize suffering.

### Study design

The mice were randomly divided into four equal groups, as follows:

- Sham group: Underwent anesthesia followed by a midline laparotomy without exposure to I/R.
- Control group: Subjected to 30 minutes of renal ischemia via artery clamping, followed by 120 minutes of reperfusion [9].
- Vehicle group: Received an intraperitoneal injection of Dimethyl sulfoxide (DMSO), the solvent for Compound 21, 30 minutes before I/R [10].
- Treatment group: Administered Compound 21 at 0.3 mg/kg intraperitoneally, 30 minutes before I/R initiation [11].

### Statistical analysis

Data analysis was performed using GraphPad Prism version 8.1, with results presented as mean ± standard error of mean (SEM). Group comparisons were made using one-way ANOVA followed by the Bonferroni correction for multiple comparisons. The Kruskal-Wallis test was used to assess histological tissue injury scores. A p-value of ≤0.05 was considered statistically significant.

## RESULTS

**Figure 1:**
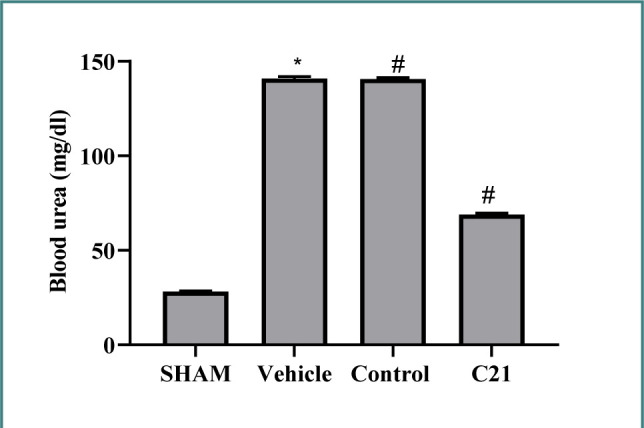
Urea (mg/dl) levels in study groups. Results are represented as mean ± standard error of mean, *p≤0.05 compared to sham, #p≤0.05 versus control.

**Figure 2:**
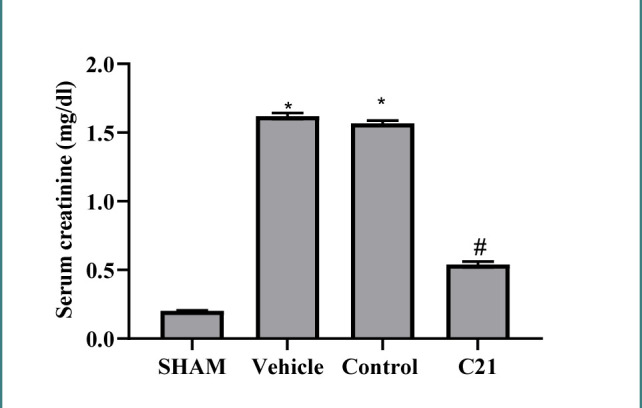
Creatinine (mg/dl) levels in study groups. Results are expressed as mean ± standard error of mean, *p≤0.05 versus sham group, #p≤0.05 versus control and vehicle groups.

The study assessed urea and creatinine levels, which are markers of kidney function, showing that urea and creatinine concentrations were higher in the ischemia/reperfusion (I/R) group and the vehicle group than in the sham group. However, pretreatment with the compound C21 significantly reduced these levels (Figures 1 and 2).

**Figure 3:**
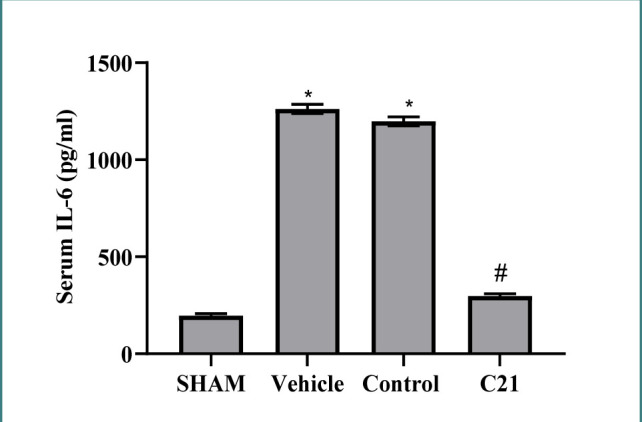
IL-6 levels (pg/ml) in study groups. Results are expressed as mean ± standard error of mean, *p≤0.05 versus sham group, #p≤0.05 versus control and vehicle groups.

After renal I/R, mice had increased serum IL-6 levels compared to the sham group, as shown in Figure 3. C21 pretreatment significantly decreased these levels, suggesting its anti-inflammatory properties.

**Figure 4:**
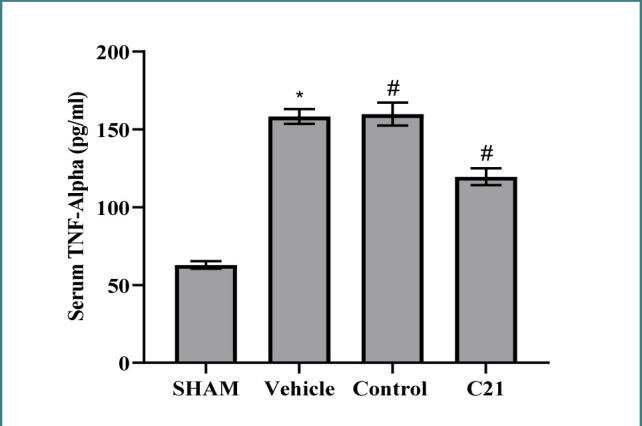
Serum levels of TNF-α (pg/ml) in study groups. Data are expressed as mean ± standard error of mean, *p≤0.05 versus the sham group, #p≤0.05 versus the control group.

Serum TNF-α levels were higher in the control and vehicle groups than in the sham groups (Figure 4). In contrast, treatment with C21 significantly decreased TNF-α levels compared to the control, demonstrating its potential therapeutic effect.

**Figure 5:**
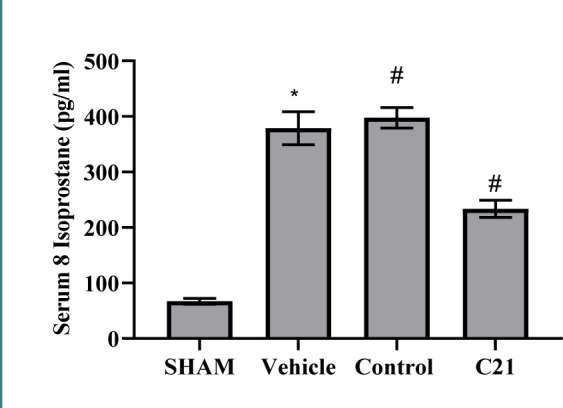
Levels of 8-isoprostane (pg/ml) in study groups. Results are expressed as mean ± standard error of mean, *p≤0.05 versus the sham group, #p≤0.05 versus the control group.

To further explore the role of oxidative stress in renal I/R, 8-isoprostane was investigated. High levels of this marker were seen in the control and vehicle groups compared to the sham group (Figure 5). Pretreatment of mice with C21 notably decreased this marker compared to the control group.

**Figure 6:**
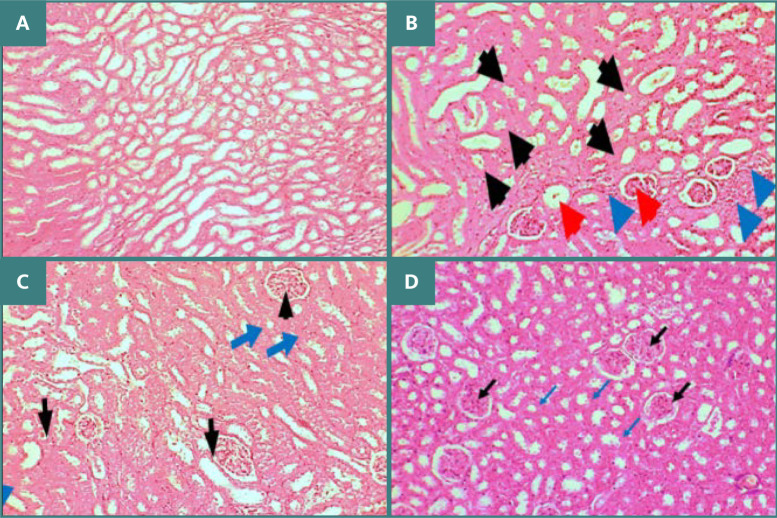
Comparative H&E staining of renal tissue sections (magnification 10X). A: Normal morphology in the sham group. B: Control group with glomerular atrophy (black arrows), tubular atrophy (blue arrows), and Bowman space dilation (red arrows). C: Vehicle group with normal glomeruli (black arrows) and severe tubular hypertrophy. D: C21 group with preserved glomerular structure (black arrows) and mild tubular hypertrophy (blue arrows).

Histological analysis was performed to explore the severity of renal damage and investigate the potential ameliorative influence of C21 using hematoxylin and eosin stain (H&E). Compared to the sham group, which showed normal tissue morphology, the control and vehicle groups had signs of renal damage, including lesions in the glomerular tuft and hypertrophy of renal tubules (Figure 6A-C). Administration of C21 mitigated renal injury featuring normal glomeruli and mild hypertrophy of renal tubules (Figure 6D).

**Figure 7:**
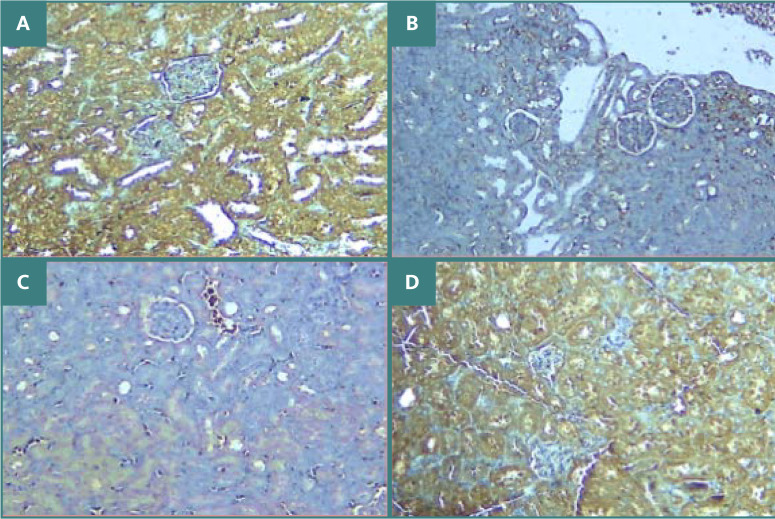
P-AKT immunostaining in renal tissues across study groups. A: Sham group with positive P-AKT staining (brown) at 100x magnification. B: The control group shows negative P-AKT (blue color) x100. C: Vehicle group shows negative P-AKT (blue color) x100. D: The C21-treated group exhibits strong P-AKT staining (brown) at x100

The immunohistochemical analysis highlighted positive phosphorylated AKT in the sham group (evidenced by brown staining), in contrast to the control and vehicle groups, which showed no such reaction (Figures 7A-C). In contrast, treatment with C21 resulted in immunoreactivity against phosphorylated AKT (Figure 7D).

**Figure 8:**
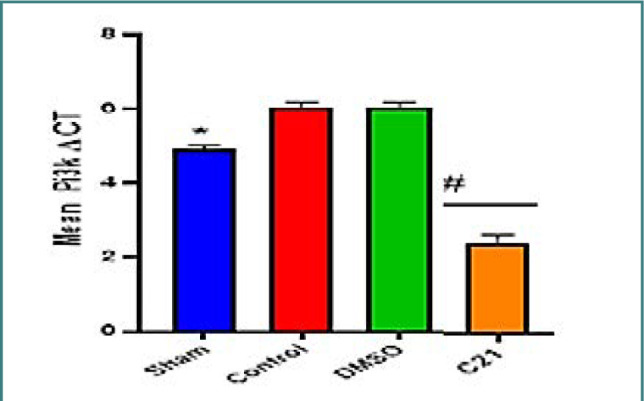
Gene expression of PI3K among the study groups, data are presented as mean SEM, *p<0.05 versus control; #p<0.05 versus control group

To further explore the AKT signaling pathway, the expression of the PI3K gene was investigated in the renal tissues. Compared to the sham group, the control and vehicle groups showed low levels of PI3K expression (Figure 8). High levels of PI3K were seen in the renal tissues of mice treated with C21 (Figure 8).

## DISCUSSION

Ischemia-reperfusion (I/R) injury is a well-recognized cause of acute kidney impairment. Recently, studies have demonstrated the critical regulatory role of the renin-angiotensin system (RAS) axis in AKI [11]. We observed high levels of TNF-α and IL-6 in the blood (p<0.05) post-I/R injury compared to the sham group.

High levels of urea and creatinine were also observed in mice subjected to I/R compared to the sham group. These results agree with Al-Jabbar *et al*., who revealed similar results of increased urea and creatinine following 30 min of ischemia and 2h of blood restoration [12]. Treatment with C21 reduced these levels, suggesting ameliorative effects. These findings align with previous studies, highlighting the renoprotective effects of AT2 receptor activation against I/R-induced kidney damage by reducing urea, creatinine, and proinflammatory cytokines [13-15].

Our study also found elevated levels of 8-isoprostane in mice subjected to I/R, consistent with literature revealing marked increases in this biological marker following I/R injury. This suggests that oxidative stress and reactive oxygen species (ROS) generation contribute to inflammation following kidney damage [16]. In contrast, low levels of 8-isoprostane were observed in mice treated with C21.

These results are consistent with prior research indicating that AT2 receptor activation reduced oxidative stress and mitigated kidney injury, improving renal function in obese rats on a high-sodium diet [17]. The morphological examination of renal tissues in mice subjected to ischemia/reperfusion (I/R) demonstrated significant tissue damage, evidenced by the dilation of renal tubules, vacuolization, loss of brush border, and glomerular changes. These findings are in line with previous studies [18-20]. Pretreatment with C21 reduced renal tissue damage, characterized by mild injury and a corresponding low score. These results support a previous study that revealed that C21 reduced necrotic lesions and preserved tubular morphology [21]. The present study found that the expression levels of phosphorylated AKT and PI3K were lower in I/R mice compared to the sham group. This aligns with prior research indicating that the PI3K-AKT signaling pathway plays a prominent role in the pathology of renal I/R injury [22]. Treatment with C21 resulted in marked increases in phosphorylated AKT and PI3K. This is consistent with previous data showing that activation of AT2 receptors enhances the production of nitric oxide via the PI3K-AKT pathway [23].

## CONCLUSION

This study identified that treatment with C21 reduced proinflammatory cytokines and oxidative stress. Furthermore, C21 treatment increased the levels of phosphorylated AKT and PI3K in renal tissues and improved renal function and tissues following I/R injury. These results suggest that C21 has ameliorative effects against renal I/R injury by interfering with the PI3K-AKT pathway.
